# Supplementary material for: Application of Artificial Intelligence Methods for Imaging of Spinal Metastasis
Source: Cancers (Basel). 2022 Aug 20;14(16):4025. doi: 10.3390/cancers14164025 (PMC9406500; doi:10.3390/cancers14164025)
Supplement: Supplementary file 1 [file cancers-14-04025-s001.zip › cancers-1832468-supplementary.pdf]

Supplementary Figure S1.

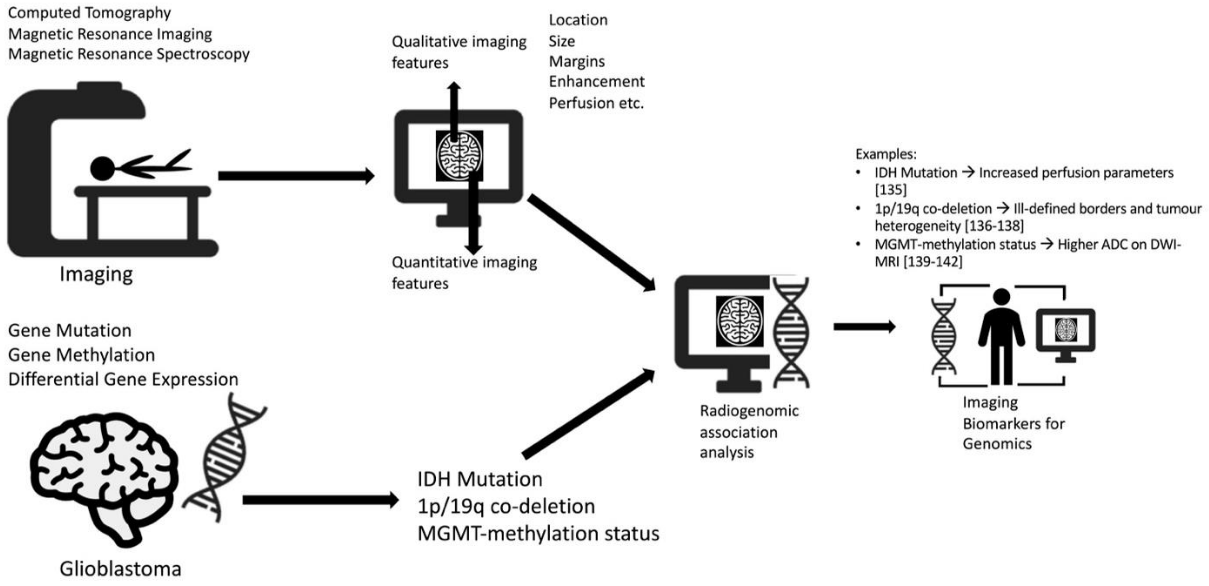

Supplementary Figure S1. Diagram showing an example of radiogenomics framework for glioblastoma (brain tumour), how imaging biomarkers are derived and used to predict genomics such as IDH-mutation [135], 1p/19q co-deletion[136-138] and MGMT-methylation status [139-142].
